# Inhibitory effects of prostaglandin E_2_ on collagen synthesis and cell proliferation in human stellate cells from pancreatic head adenocarcinoma

**DOI:** 10.1186/1471-2407-14-413

**Published:** 2014-06-09

**Authors:** Ewa Pomianowska, Dagny Sandnes, Krzysztof Grzyb, Aasa R Schjølberg, Monica Aasrum, Ingun H Tveteraas, Vegard Tjomsland, Thoralf Christoffersen, Ivar P Gladhaug

**Affiliations:** 1 Institute of Clinical Medicine, Faculty of Medicine, University of Oslo, Oslo, Norway; 2 Department of Hepato-pancreato-biliary Surgery, Oslo University Hospital, Rikshospitalet, PO Box 4956, Nydalen 0424 Oslo, Norway; 3 Department of Pharmacology, Faculty of Medicine, University of Oslo and Oslo University Hospital, Oslo, Norway; 4 Department of Pathology, Oslo University Hospital, Rikshospitalet, Oslo, Norway

**Keywords:** Pancreatic stellate cells, Prostaglandin E_2_, Cyclic AMP, DNA synthesis, Collagen synthesis

## Abstract

**Background:**

Several studies have described an increased cyclooxygenase-2 (COX-2) expression in pancreatic cancer, but the role of COX-2 in tumour development and progression is not clear. The aim of the present study was to examine expression of COX-2 in cancer cells and stromal cells in pancreatic cancer specimens, and to explore the role of PGE_2_ in pancreatic stellate cell proliferation and collagen synthesis.

**Methods:**

Immunohistochemistry and immunofluorescence was performed on slides from whole sections of tissue blocks using antibodies against COX-2 and α-smooth muscle actin (αSMA). Pancreatic stellate cells (PSC) were isolated from surgically resected tumour tissue by the outgrowth method. Cells were used between passages 4 and 8. Collagen synthesis was determined by [^3^H]-proline incorporation, or by enzyme immunoassay measurement of collagen C-peptide. DNA synthesis was measured by incorporation of [^3^H]-thymidine in DNA. Cyclic AMP (cAMP) was determined by radioimmunoassay. Collagen 1A1 mRNA was determined by RT-qPCR.

**Results:**

Immunohistochemistry staining showed COX-2 in pancreatic carcinoma cells, but not in stromal cells. All tumours showed positive staining for αSMA in the fibrotic stroma. Cultured PSC expressed COX-2, which could be further induced by interleukin-1β (IL-1β), epidermal growth factor (EGF), thrombin, and PGE_2_, but not by transforming growth factor-β1 (TGFβ). Indirect coculture with the adenocarcinoma cell line BxPC-3, but not HPAFII or Panc-1, induced COX-2 expression in PSC. Treatment of PSC with PGE_2_ strongly stimulated cAMP accumulation, mediated by EP2 receptors, and also stimulated phosphorylation of extracellular signal-regulated kinase (ERK). Treatment of PSC with PGE_2_ or forskolin suppressed both TGFβ-stimulated collagen synthesis and PDGF-stimulated DNA synthesis.

**Conclusions:**

The present results show that COX-2 is mainly produced in carcinoma cells and suggest that the cancer cells are the main source of PGE_2_ in pancreatic tumours. PGE_2_ exerts a suppressive effect on proliferation and fibrogenesis in pancreatic stellate cells. These effects of PGE_2_ are mediated by the cAMP pathway and suggest a role of EP2 receptors.

## Background

Pancreatic adenocarcinoma is one of the most lethal cancers of all solid malignancies with a 5 year survival of less than 5% [1-3]. A particular feature of primary pancreatic adenocarcinoma is the extensive fibrotic stromal reaction known as tumour desmoplasia surrounding these tumours [4-6]. There is increasing evidence that stromal cells are of major importance for tumour progression, by interacting in many ways with the malignant cells, such as reciprocal paracrine proliferative stimulation and angiogenesis, contributing to the early invasive growth and metastasis of this tumour [6]. These observations have raised the possibility that targeting the stromal cells to interrupt paracrine stromal signalling mechanisms may represent a new treatment strategy in pancreatic cancer. Animal studies have also indicated that targeting the tumour stroma of pancreatic cancer may improve drug delivery [7-9].

Multiple lines of evidence suggest that pancreatic stellate cells (PSC) have a major role in the development of pancreatic cancer desmoplasia [4-6,10]. These cells, which are normally quiescent cells in the pancreas, are induced during pancreatic injury to undergo transformation into a myofibroblast-like phenotype expressing alpha smooth muscle actin (αSMA). Studies of human and rat PSC in culture have identified a number of growth factors, cytokines, and hormones as regulators of pancreatic stellate cell activation [6]. Activation promotes PSC proliferation, migration, and extracellular matrix (ECM) deposition.

Overexpression of COX-2 has been reported in a number of epithelial cancers, including pancreatic cancer [11-16]. Transgenic mouse models have suggested that COX-2 overexpression in pancreatic ductal cells contributes to pancreatic tumour development [17,18]. Upregulation of COX-2 leads to increased production of prostaglandins, in particular PGE_2_. PGE_2_ may affect both cancer cells and different stromal cells through its effects on EP and FP receptors [19,20]. While EP2 and EP4 receptors are G_s_-coupled receptors that stimulate adenylyl cyclase activity, EP3 receptors are G_i_-coupled and inhibit adenylyl cyclase activity. EP1 receptors elevate the intracellular Ca^2+^-levels through mechanisms that may involve both phospholipase C-dependent and independent mechanisms [19-21], and FP receptors are G_q_-coupled and elevate intracellular Ca^2+^-levels [19,20]. In addition, several of these receptors may signal via G protein-independent mechanisms [22].

Fibroblasts may be stimulated by PGE_2_. Elevation of the intracellular level of cAMP in response to PGE_2_ or other stimuli in fibroblasts from different tissues has been found to limit their proliferation, migration, and collagen secretion, as well as the differentiation of fibroblasts to myofibroblasts [23-25]. These effects appear to be mediated via EP2 and EP4 receptors. It has also been reported that PGE_2_ may promote fibroblast proliferation through activation of EP1, EP3, or FP signalling [26-29]. In hepatic stellate cells, PGE_2_ has been found to inhibit transforming growth factor β (TGFβ)-mediated induction of collagen mRNA [30], as well as proliferation induced by platelet-derived growth factor (PDGF) or thrombin [31,32]. However, the role of PGE_2_ in pancreatic fibrosis is not well known. The aim of the present study was to examine further the effects of PGE_2_ on pancreatic stellate cell proliferation and collagen synthesis.

## Methods

### Patients

The study protocol and patient consent documents were approved by the Regional Committee for Medical and Health Research Ethics (REC South East, project number S-05081), and was in compliance with the Helsinki Declaration. Written informed consent was obtained from all study participants. The study included only adults.

### Chemicals

Dulbecco’s Modified Eagle’s Medium, Ham’s F12 medium, RPMI 1640 medium, glutamine, and Pen-Strep (10.000 U/ml) were obtained from Lonza (Verviers, Belgium). HEPES, amphotericin, and heat-inactivated fetal bovine serum (FBS) was purchased from Gibco (Grand Island, NY, USA). Epidermal growth factor (EGF), adenosine 3’:5’-cyclic monophosphate (cAMP), 3-isobutyl 1-methylxanthine (IBMX), L-ascorbic acid, and 3-aminopropionitrile fumarate salt were obtained from Sigma-Aldrich (St.Louis, MO, USA). Human platelet derived growth factor (PDGF), recombinant human transforming growth factor-β (TGF-β), and recombinant human interleukin-1β (IL-1β) were obtained from R&D Systems Europe, Ltd (Abingdon, England). Recombinant interleukin-1 receptor antagonist (Anakinra®) was a gift from Swedish Orphan Biovitrum AS, [6-^3^H] thymidine (20–30 Ci/mmol), [2,8-^3^H] adenosine 3’,5’-cyclic phosphate ammonium salt (33.0 Ci/mmol), and L-[2,3-^3^H] proline (55.0 Ci/mmol) were purchased from PerkinElmer (Boston, MA, USA). L161982 (N-[[4’-[[3-butyl-1,5-dihydro-5-oxo-1-[2-(trifluoromethyl)phenyl]-4 H-1,2,4-triazol-4-yl]methyl][1,1'-biphenyl]-2-yl]sulfonyl]-3-methyl-2-thiophenecarboxamide, AH6809 (6-isopropoxy-9-oxoxanthene-2carboxylic acid), and prostaglandin E_2_ (PGE_2_) were obtained from Cayman Chemical (Ann Arbor, MI, USA). Procollagen Type I C-peptide enzyme immunoassay kit was purchased from Takara Bio Inc., Japan. All other chemicals were of analytical quality. Antibodies against phosphorylated Akt^Ser473^, total Akt, dually phosphorylated ERK^Thr202/Tyr204^, and GAPDH were obtained from Cell Signaling Technology (Boston, MA, USA). Antibodies against COX-2 were obtained from Cayman Chemical (Ann Arbor, MI, USA) or from Thermo Fischer Scientific Inc (Fremont, CA, USA). Anti-ERK antibody was from Upstate/Millipore (Billerica, MA, USA). Antibodies against TGF-β receptor II and PDGF receptor β were purchased from Cell Signaling Technology (Boston, MA, USA). Antibody against EP2 receptor was obtained from Cayman Chemical (Ann Arbor, MI, USA). Secondary antibodies were purchased from Bio-Rad Laboratories (Hercules, CA, USA). Antibodies against vimentin and cytokeratins 7 and 19 were provided by DAKO (Glostrup, Denmark).

### Isolation and culture of human pancreatic stellate cells

Human pancreatic stellate cells (PSC) were isolated by the outgrowth method developed by Bachem et al. [33]. Pancreatic tissue blocks (100–150 mg) were obtained during pancreatic surgery from patients with resectable pancreatic head adenocarcinoma. Altogether, stellate cell cultures were established from a total of 20 different patients. Briefly, the tissue blocks were cut using a razor blade (0.5–1 mm^3^) and seeded in 10 cm^2^ uncoated culture wells (6 per plate; 3–5 pieces per well) in a 1:1 (vol/vol) mixture of Dulbecco’s modified Eagle medium (DMEM) with Ham’s F12 medium, supplemented with l-glutamine (2 mmol/L), 100 U/ml Pen-Strep, 2.5 μg/ml amphotericin, and 10% FBS. Tissue blocks were cultured at 37°C in a 5% CO_2_/air humidified atmosphere. Twenty-four hours after seeding, the small tissue blocks were transferred to new culture plates. Culture medium was changed every third day. The PSCs grew out from the tissue blocks 7 to 10 days later. The small tissue blocks were removed after 2–3 weeks. After reaching confluence, monolayers were trypsinized and passaged 1:3. The purity of the cells was assessed by morphology (most cells were stellate-like, with long cytoplasmatic extensions; some were also spindle shaped) and cytofilament staining of αSMA and vimentin. None of the cells were positive for cytokeratins 7 or 19 (data not shown). All experiments were performed using cell populations between passage 4 and 8.

### Pancreatic adenocarcinoma cell lines

BxPC-3, HPAFII, and Panc-1 pancreatic adenocarcinoma cell lines were purchased from ATCC (Manassas, VA, USA). BxPC-3 cells were cultured in RPMI medium containing 4.5 g/l glucose, HPAFII cells were cultured in Dulbecco’s modified Eagle’s medium containing 1 g/l glucose, and Panc-1 cells were cultured in Dulbecco’s modified Eagle’s medium containing 4.5 g/l glucose. The media were supplemented with glutamine (2 mM, or 4 mM in the case of Panc-1), 100 U/ml Pen-Strep, and 10% fetal bovine serum (FBS). Cells were plated in Transwell® inserts (Corning Incorporated, Corning, NY, USA) at a density of 100.000/cm^2^ in serum-containing medium and cultured overnight. The next day, medium was replaced with fresh, serum-free medium, and cells were cultured overnight. The following day, the Transwells were transferred to 12 well Costar plates containing stellate cells in the lower compartment, and cells were cocultured for 48 hours.

### Coculture of pancreatic stellate cells with pancreatic adenocarcinoma cell lines

Pancreatic stellate cells were plated at a density of 10.000 cells/cm^2^ in 12 well Costar plates with serum-containing medium and cultured overnight. The following day, medium was replaced with fresh, serum-free medium, and cells were cultured overnight. The next day, the serum-free medium was changed, and Transwells containing pancreatic adenocarcinoma cell lines were placed on top. Cells were cocultured for 48 hours before harvesting for immunoblotting.

### Measurement of DNA synthesis

Pancreatic stellate cells were seeded into 12 well Costar plates at a density of 10.000 cells/cm^2^ in serum-containing medium and cultured overnight. On the following day, medium was replaced with fresh, serum-free medium. The next day, the serum-free medium was changed 30 minutes before addition of agonists. The cells were harvested after pulsing for 6 hours with [^3^H]thymidine (18–24 hours after addition of agonists), and DNA synthesis was measured as the amount of radioactivity incorporated into DNA as previously described [34]. Briefly, medium was removed, and cells were washed twice with 0.9% NaCl. The cellular material was dissolved with 1 ml 0.5 N NaOH for 3 hours at 37°C, collected, mixed with 1 ml H_2_O, and precipitated with 0.5 ml 50% trichloroacetic acid (TCA). The acid-precipitable material was transferred to glass fiber filters (GF/C Whatman, GE Healthcare, UK) and washed twice with 5.0 ml 5% TCA, followed by liquid scintillation counting of the filters in a Packard Tri-Carb 1900 TR liquid scintillation counter.

### Measurement of collagen synthesis

Collagen synthesis was assessed by quantification of [^3^H] proline incorporation into acetic acid-soluble proteins as described by Jaster et al. [35]. Pancreatic stellate cells were plated in 24 well Costar plates at a density of 10.000 cells/cm^2^ in serum-containing medium and cultured overnight. The following day, medium was replaced with fresh, serum-free medium. The next day, serum-free medium was changed, and agonists and/or antagonist were added. After 24 hours, the medium was replaced with fresh serum-free medium containing 100 μg/ml ascorbic acid, 100 μg/ml 3-aminopropionitrile, and 2 μCi/ml [^3^H] proline, and fresh agonists were added. The reaction was stopped 24 hours later, by addition of 50 μl/ml 10 N acetic acid. After an overnight incubation at 4°C, culture supernatants were transferred to microcentrifuge tubes, mixed with 100 μl/ml FBS, 5 μg/ml rat tail collagen and 250 μl/ml 25% NaCl dissolved in 0.5 N acetic acid, and incubated at 4°C for 30 minutes. Protein precipitates collected by centrifugation (30 min, 10,000 g) were washed twice with 5% NaCl, followed by dissolution of the pellet in 0.5 N acetic acid. [^3^H] proline incorporation was determined by liquid scintillation counting in a Packard Tri-Carb 1900 TR scintillation counter. In initial experiments, collagen synthesis was determined in parallell samples by measurement of procollagen type I C-peptide by an enzyme immunoassay. The two methods yielded similar results (data not shown).

### RNA extraction and real-time quantitative RT-qPCR

Pancreatic stellate cells were plated at a density of 10.000/cm^2^ in 20 cm^2^ wells in serum-containing medium and cultured overnight. On the following day, medium was replaced with serum-free medium. The next day the medium was changed 30 minutes before agonists and/or antagonist were added, as indicated. The cells were stimulated for 24 hours. Total RNA was prepared from the samples using RNA Easy Mini kit (Qiagen Inc, Valencia, CA, USA) and cDNA was synthesized with SuperScript III Reverse Transcriptase First-Strand cDNA Synthesis kit according to the manufacturer’s protocol (InVitrogen, Carlsbad, CA, USA). Quantitative PCR was performed with Platinum SYBR Green Master Mix (Life Technologies, Oslo, Norway) on 7900 Real-Time PCR system with 7900 System SDS 2.3 Software (Applied Biosystems) according to the manufacturer’s protocol. Specific primers for collagen 1A1 were: forward, 5’-TGACGTGATCTGTGACGAGAC-3’ and reverse, 5’- GGTTTCTTGGTCGGTGGGT −3’ (Life Technologies Oslo, Norway). Glyceraldehyde-3-phosphate dehydrogenase (GAPDH) was utilized as housekeeping gene, and specific primers were: forward, 5’-CCACCATGGAGAAGGCTGGGGCTC-3’ and reverse 5’-AGTGATGGCATGGACTGTGGTCAT3’ (Life Technologies, Oslo, Norway). The primers were designed using Primer-BLAST [36]. All reactions were performed in triplicates including non-template controls. The results were analyzed using the ΔΔCt method [37]. Results for collagen 1A1 were normalized to GAPDH, and controls were assigned a value of 100%.

### Cyclic AMP measurement

Pancreatic stellate cells were plated in 12 well Costar wells at a density of 10.000 cells/cm^2^ in serum-containing medium. On the following day, medium was replaced with fresh, serum-free medium. The next day, medium was replaced with Krebs-Ringer-Hepes buffer, pH 7.4, containing 10 mM glucose. After preincubation for 30 minutes, cells were stimulated with PGE_2_ or forskolin as indicated in the figure legends. The reaction was stopped by removing the buffer and adding 5% TCA. cAMP in the neutralized TCA extract was determined by radioimmunoassay as previously described [38].

### Immunoblotting

Aliquots with approximately 7000 cells (total cell lysate prepared in Laemmli buffer) were electrophoresed on 12% (w/v) polyacrylamide gels (acrylamide: N’N’-bis-methylene acrylamide 30:1). This was followed by protein electrotransfer to nitrocellulose membranes and immunoblotting with antibodies against phospho-Akt, total Akt, phospho ERK1/2, total ERK, COX-2, and GAPDH, respectively. Immunoreactive bands were visualized with enhanced chemiluminescence using LumiGLO (KPL Protein research Products, Gaithersburg, MD, USA).

### Immunohistochemistry

Formalin-fixed, paraffin-embedded tissues from pancreatectomy specimens were sectioned (3 μm), and dried at 60°C. Further processing was carried out in the Ventana BenchMark Ultra machine (Ventana Medical Systems Inc. (Tucson Arizona USA) according to the manufacturer’s recommendations. Slides were incubated with monoclonal anti-COX-2 antibodies (Thermo Fischer Scientific rabbit), Universal Alkaline Phosphatase Red Detection Kit (Ultra View 760–501) and a-SMA (Dako M.0851, DAB (Ultra View 760–500). Finally, slides were counterstained with haematoxylin, fixed, mounted and analyzed using an inverted light microscope (Olympus, Center Valley, PA, USA).

### Immunofluorescence staining

Immunofluorescence staining was performed to examine COX-2 expression in the tumour slides. Formalin-fixed, paraffin-embedded tissues from pancreatectomy specimens were sectioned (3 μm), dried at 60°C and hydrated. Slides were incubated with monoclonal anti-COX-2 antibody (Thermo sp21 rabbit) and anti-αSMA (DAKO 1A4 mouse) for 30 min at room temperature in Ventana diluents. After washing with PBS, slides were incubated with secondary antibody conjugates (Alexa 555 anti-rabbit and Alexa 488 anti - mouse) in the dark for 1 hour in Dako diluents. After three washes with PBS, slides were mounted in VECTASHIELD containing DAPI (Vector Laboratories Inc., Burlingame, CA, USA). Fixed cells were observed under a fluorescence microscope.

Immunofluorescence staining was also performed on the cultured pancreatic stellate cells. Cells were first seeded into a Lab-Tek®II Chamber Slide™ System (Nunc International, Naperville, IL, USA) and were cultured for 24 hours before they were fixed in 4% paraformaldehyde at room temperature for 15 minutes. Cells were then washed three times and incubated with 5% BSA for 30 minutes to block non-specific binding. Slides were further processed as describe for tumour tissue.

### Statistical analyses

Results are presented as mean ± standard error of the mean (S.E.M). DNA and collagen synthesis data were analyzed by one-way ANOVA, and post test using Bonferroni correction to compare groups, using GraphPad Prism (version 5.01, GraphPad Software, San Diego, CA, USA).

## Results

### COX-2 expression in pancreatic cancer cells

**Figure 1:**
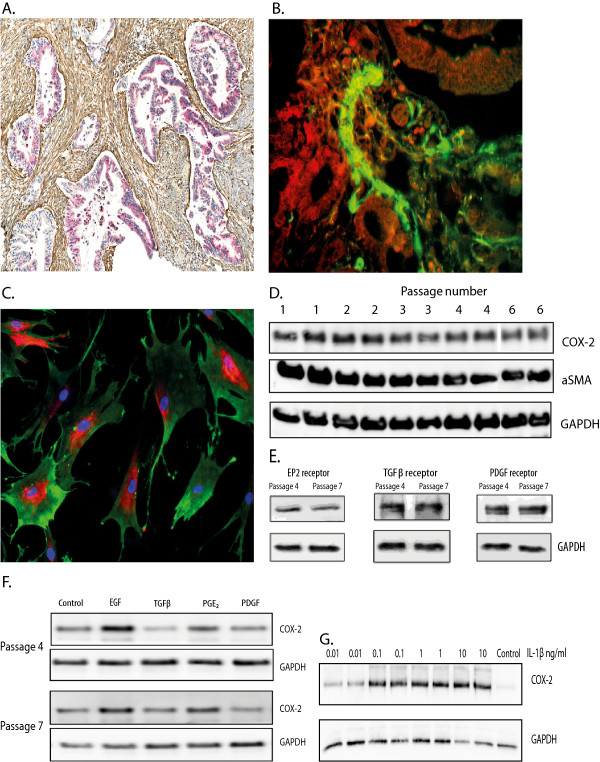
**COX-2 expression in formalin-fixed, paraffin-embedded tumour tissue from pancreatic cancer and isolated pancreatic stellate cells. A.** Immunohistochemistry of COX-2 expression in tumour tissue from pancreatic cancer. COX-2 positive cells - red colour, fibrotic stroma αSMA positive - brown colour. **B.** Immunofluorescence of COX-2 expression in tumour tissue from pancreatic cancer. COX-2 positive cells - red colour, stroma αSMA positive - green colour. **C.** Immunofluorescence staining of cultured pancreatic stellate cells, passage five; COX-2 positive cells - red colour, αSMA positive cells - green colour, nucleus -blue colour. **D.** Expression of COX-2 and αSMA in different cell passage numbers. **E.** Expression of EP2 receptors, TGFβ receptors and PDGF receptors in two different cell passages **F.** Induction of COX-2 protein by EGF (10 nM), TGFβ (10 ng/ml), PGE_2_ (10 uM), and PDGF (10 ng/ml) in two different cell passages. Cells in serum-free medium were stimulated with agonists for 24 hours before cells were harvested and lysates examined by Western blots as described in Methods. Results are from one representative experiment of four. **G.** Concentration dependent induction of COX-2 protein by IL-1β. Cells were stimulated in serum-free medium for 24 hours. Results are from one typical experiment of three.

COX-2 expression in tumour tissue from pancreatic cancer was examined by double staining immunohistochemistry for COX-2 and αSMA. The cancer cells generally exhibited strong COX-2 staining (Figure 1A). We also found strong αSMA staining in the tumour stroma, indicating the presence of activated PSC. However, we could not detect double staining with COX-2 and αSMA in the stroma (Figure 1A). This was examined further by immunofluorescence, which failed to detect any COX-2 staining in the stroma (Figure 1B).

### COX-2 expression in cultured human PSC

**Figure 2:**
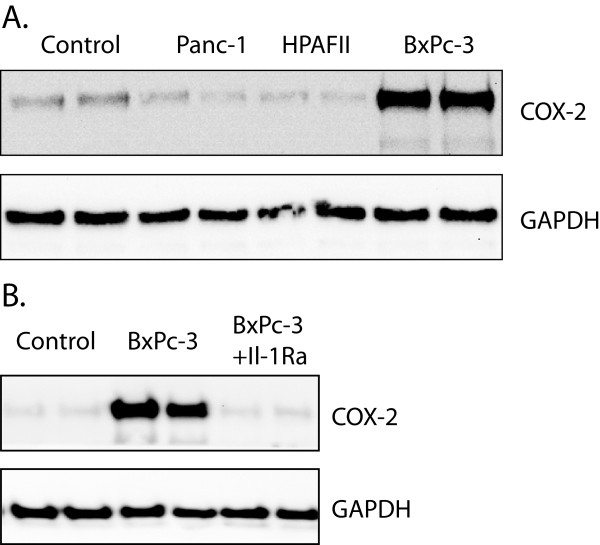
**Induction of COX-2 protein in pancreatic stellate cells by indirect coculture with pancreatic adenocarcinoma cell lines. A.** Effect of coculture with Panc-1, HPAFII, and BxPC-3 cells. Cells were cocultured in serum-free medium for 48 hours, before harvesting and analysis by Western blots as described in Methods. Results are from one typical of three experiments. **B.** Inhibition of COX-2 induction by coculture with BxPC-3 cells when stellate cells were pretreated with IL-1 receptor antagonist (1 μg/ml) for 1 hour before coculture for 48 hours. Results are from one typical of four experiments.

During culture of PSC, immunofluorescence staining in different passages revealed perinuclear staining with the COX-2 antibody in cells that were αSMA positive (Figure 1C). The expression of COX-2, αSMA, EP2 receptors, TGFβ receptors and PDGF receptors was found to be stable as a function of cell passage number as assessed by Western blotting (Figure 1D, 1E). Treatment of PSC with EGF and PGE_2_ increased the expression of COX-2, whereas treatment with TGFβ did not. This expression pattern was observed in cells of both low and high passage numbers (Figure 1F). PDGF had no significant effect. Thrombin also induced COX-2 expression (data not shown). Interleukin-1β (IL-1β) was found to be a potent inducer of COX-2 expression, with maximal induction obtained at 0.1 ng/ml (Figure 1G). Coculture of pancreatic adenocarcinoma cell lines with pancreatic stellate cells was previously found to upregulate COX-2 mRNA in both stellate cells and adenocarcinoma cell lines [39]. We examined the effect of coculture of stellate cells with the adenocarcinoma cell lines BxPC-3, Panc-1, and HPAFII. Of these, only BxPC-3 cells induced COX-2 protein in the stellate cells (Figure 2A). Furthermore, this effect was abolished when the stellate cells were pretreated for one hour with an IL-1 receptor antagonist (Figure 2B).

### PGE_2_ stimulates EP2-mediated cAMP accumulation in PSC

**Figure 3:**
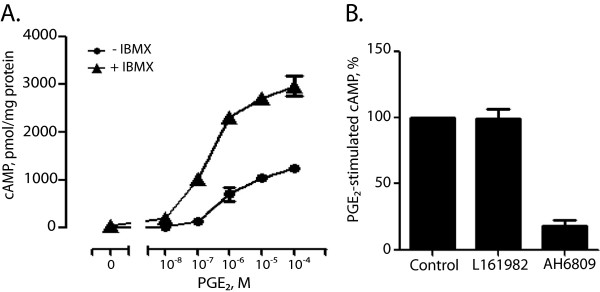
**PGE**_**2**_**-stimulated cAMP accumulation in pancreatic stellate cells. A.** Dose-dependent effect of PGE_2_ in the absence and presence of 0.5 mM isobutylmethylxanthine (IBMX). Cells were cultured as described in Methods, and were stimulated for 5 minutes. Results are presented as mean + S.E.M. of three replicates from one representative of three experiment. **B.** Effect of EP4 receptor antagonist (L161982, 10 μM) and EP2 receptor antagonist (AH6809, 30 μM) on PGE_2_-stimulated cAMP accumulation (1 μM PGE_2_). Cells were preincubated with antagonists for 30 minutes before stimulation with PGE_2_ for 15 minutes in the presence of 0.5 mM IBMX. Results are presented as mean ± S.E.M. of five experiments.

PGE_2_ may affect cells through both EP and FP receptors. Because fibroblasts from different tissues have been found to express mainly EP2 and EP4 receptors [24,40,41], we examined the effect of PGE_2_ on cAMP accumulation in the stellate cells. When stellate cells were stimulated for 5 min with 100 μM PGE_2_ or 50 μM forskolin, a direct activator of adenylyl cyclase [42], in the presence of the phosphodiesterase inhibitor isobutylmethylxanthine (IBMX), cAMP levels were elevated 16.8 ± 5.8-fold (mean ± S.E.M.) above basal levels with PGE_2_, and 33.0 ± 11.7-fold above basal with forskolin (n = 7). PGE_2_ induced a strong, dose-dependent accumulation of cAMP, both in the absence and presence of IBMX (Figure 3A). When cells were preincubated with the EP4 receptor antagonist L-161982 [43], no significant inhibition of PGE_2_-stimulated cAMP accumulation was observed. In contrast, AH6809, which is commonly used as en EP2 receptors antagonist [19], almost abolished the cAMP response, suggesting that cAMP accumulation in these cells is mediated mainly by EP2 receptors (Figure 3B).

### PGE_2_ inhibits DNA synthesis in PSC

**Figure 4:**
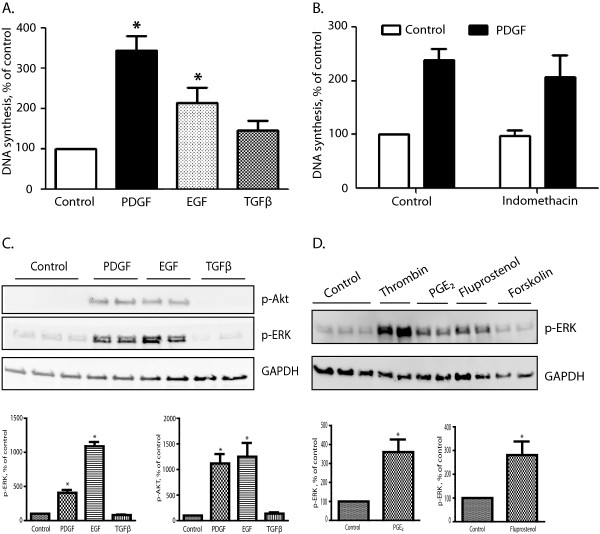
**Effect of different agonists on DNA synthesis and phosphorylation of ERK and Akt in pancreatic stellate cells. A.** Effect of PDGF (10 ng/ml), EGF (10 nM), and TGFβ (10 ng/ml) on DNA synthesis. Cells in serum-free medium were stimulated for 24 hours, with [^3^H] thymidine added at 18 hours. DNA synthesis was assessed as described in Methods. Results are presented as mean +/−SEM of six experiments. **B.** Effect of pretreatment with indomethacin (10 μM) for one hour before stimulation of cells with PDGF for 24 hours. Results are presented as mean +/−SEM of three experiments. **C.** Effect of PDGF (10 ng/ml), EGF (10 nM), and TGFβ (10 ng/ml) on phosphorylation of Akt and ERK. Cells in serum-free medium were stimulated for 5 minutes before harvesting and analysis of cell lysates on Western blots. Blots are from one typical of four experiments. Histograms represent mean +/−SEM of four experiments. **D.** Effect of thrombin (1 U/ml), PGE_2_ (10 μM), fluprostenol (10 μM), and forskolin (10 μM) on ERK phosphorylation. Cells were stimulated for 5 minutes before harvesting. Blots from one typical of four experiments are shown. Histograms represent mean +/−SEM of four experiments. * Sign. different from control.

We next examined how PGE_2_ affected stellate cells proliferation. In agreement with previous studies [6,44,45], PDGF strongly stimulated DNA synthesis (Figure 4A). Epidermal growth factor (EGF) also stimulated DNA synthesis, although to a lesser extent than PDGF, whereas TGFβ had non-significant effect. (Figure 4A). In agreement with these findings, PDGF and EGF, but not TGFβ, significantly stimulated phosphorylation of both ERK and Akt in the stellate cells (Figure 4C). Interestingly, PGE_2_, the FP selective receptor agonist fluprostenol, and thrombin also stimulated ERK phosphorylation in the stellate cells (Figure 4D), while they did not induce Akt phosphorylation (data not shown). The effect of PGE_2_ and fluprostenol on ERK phosphorylation did not seem to involve cAMP, since forskolin did not stimulate ERK phosphorylation.

In human hepatic stellate cells several growth-stimulatory agents, including PDGF and thrombin, stimulate an acute PGE_2_ production, as well as a delayed induction of COX-2, and pretreatment with a COX inhibitor enhances their growth stimulatory effect [31]. We examined the effect of pretreatment with indomethacin on PDGF-stimulated DNA synthesis in the pancreatic stellate cells. These experiments showed that pretreatment with indomethacin did not affect PDGF-stimulated DNA synthesis in the pancreatic stellate cells (Figure 4B).

**Figure 5:**
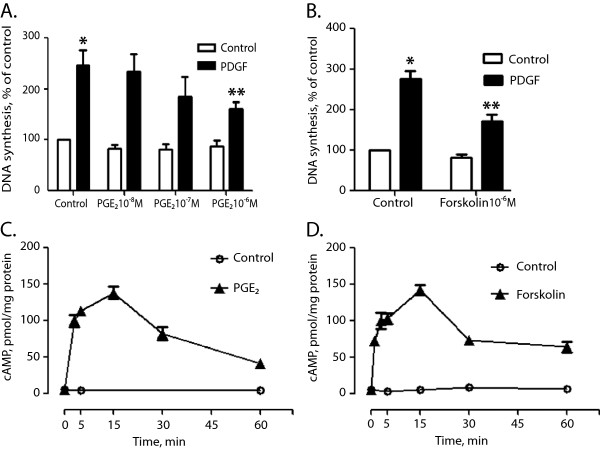
**Effect of PGE**_**2 **_**and forskolin on DNA synthesis and cAMP accumulation. A.** Effect of increasing concentrations of PGE_2_ on PDGF-stimulated DNA synthesis. Results are presented as mean ± S.E.M. of four experiments. **B.** Effect of 1 μM forskolin on PDGF- stimulated DNA synthesis. Results are presented as mean ± S.E.M. of four experiments. **C.** Time-dependent effect of 1 μM PGE_2_ on cAMP accumulation in the absence of IBMX. Results are from one typical of four experiments and are presented as mean ± S.E.M. of triplicates. **D.** Time-dependent effect of 5 μM forskolin on cAMP accumulation in the absence of IBMX. Results are from one typical of four experiments and are presented as mean ± S.E.M. of triplicates. * Sign. different from control. ** Sign. different from PDGF alone.

Treating the stellate cells with PGE_2_ did not significantly affect the basal DNA synthesis, but attenuated PDGF-stimulated DNA synthesis. PGE_2_ exerted an inhibitory effect, which was significant at a concentration of 1 μM (Figure 5A). This effect was mimicked by forskolin (Figure 5B). cAMP levels were elevated above the basal level for at least 60 minutes following stimulation with PGE_2_ (Figure 5C) or forskolin (Figure 5D). Neither fluprostenol nor thrombin had any effect on DNA synthesis, alone or in combination with PDGF (data not shown).

### PGE_2_ inhibits collagen synthesis in PSC

**Figure 6:**
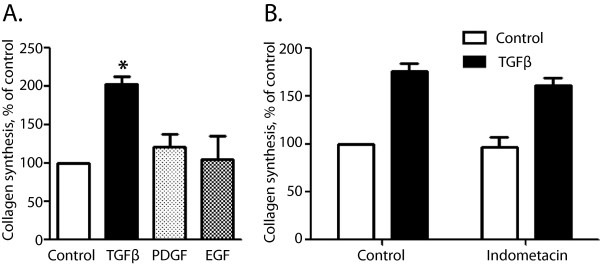
**Effects of different agonists on collagen synthesis. A.** Effect of TGFβ (10 ng/ml), PDGF (10 ng/ml) and EGF (10 nM) on collagen synthesis. Cells were cultured and stimulated with agonists for 48 hours, as described in Methods. [^3^H] proline was present for the last 24 hours of stimulation. Collagen was precipitated and radioactivity in collagen was determined as described in Methods. Results are presented as mean ± S.E.M. of five experiments. **B.** Effect of pretreatment with indomethacin (10 μM) for one hour before stimulation of cells with TGFβ for 48 hours. Results are presented as mean ± S.E.M. of three experiments. * Significantly different from control.

**Figure 7:**
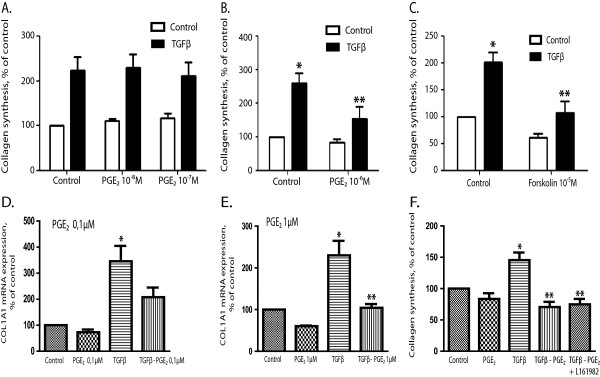
**Effect of PGE2 and forskolin on TGFβ-stimulated collagen synthesis. A, B.** Effects of different concentrations of PGE_2_ on TGFβ-stimulated collagen synthesis. Cells were stimulated for 48 hours. Results are presented as mean ± S.E.M. of six experiments. **C.** Effect of forskolin on TGFβ stimulated collagen synthesis. Cells were stimulated for 48 hours. Results are presented as mean ± S.E.M of five experiments. **D.** Effect of TGFβ (10 ng/ml) alone and in combination with 100 nM PGE_2_ on collagen 1A1 mRNA expression. Cells were stimulated for 24 hours before RNA was isolated and real time quantitative RT-qPCR was performed. Results are presented as mean ± S.E.M. of eight experiments. **E.** Effect of TGFβ (10 ng/ml) alone and in combination with 1 μM PGE2 on collagen 1A1 mRNA expression. Cells were stimulated for 24 hours before RNA was isolated and real time quantitative RT-qPCR was performed. Results are presented as mean + S.E.M. of three experiments. * Sign. different from control ** Sign. different from TGFβ alone. **F.** Effect of the EP4 receptor antagonist ( L161982 1 μM) on PGE_2_ influence on TGFβ-stimulated collagen synthesis. Cells were stimulated for 48 hours. Results are presented as mean ± S.E.M. of three experiments. *Sign. different from control ** Sign. different from TGFβ alone.

In agreement with previous findings [6,45,46] treating the stellate cells with TGFβ enhanced collagen synthesis, whereas PDGF or EGF did not significantly affect collagen synthesis (Figure 6A). In agreement with the lack of induction of COX-2 by TGFβ in the stellate cells, pretreatment with indomethacin did not affect TGFβ-induced collagen synthesis (Figure 6B). Both PGE_2_ and forskolin inhibited TGFβ-stimulated collagen synthesis, suggesting that this was a cAMP-mediated effect (Figure 7A, B, C). While we were preparing this manuscript, Charo et al. reported that PGE_2_ stimulated the mRNA expression of collagen 1A1 in an immortalized human pancreatic stellate cell line [40]. To examine this further, RNA was extracted from cultured pancreatic stellate cells and assessed for elevated gene expression of collagen 1A1 by real time RT-qPCR. While TGFβ increased gene expression, PGE_2_ alone showed a slight inhibitory effect, and significantly attenuated TGFβ-stimulated increase in gene expression of collagen 1A1 at a concentration of 1 μM (Figures 7D, 7E). Since PGE_2_ might elevate cAMP levels through EP2 or EP4 receptors, we examined the effect of EP2 and EP4 receptor antagonists on collagen synthesis. We found that the EP4 receptor antagonist L161982 did not abrogate the effect of PGE_2_ on TGFβ-induced collagen synthesis (Figure 7F) whereas results with the EP2 receptor antagonist AH 6809 were not conclusive (data not shown).

## Discussion

In the present study we have demonstrated that PGE_2_ inhibits both collagen and DNA synthesis in human pancreatic stellate cells from pancreatic adenocarcinoma. These effects are mediated by increased cAMP production. It is well known that in fibroblasts from lung and other tissues, PGE_2_ inhibits proliferation by activating G_s_-coupled EP2 and/or EP4 receptors [23-25,41,47,48]. Since EP4 inhibition affected neither the cAMP response nor the effect on collagen synthesis by PGE_2_ in our study, it is most likely that EP2 receptors mediate these inhibitory effects of PGE_2_ on cAMP and collagen synthesis. However, due to inconclusive results with the EP2 receptor antagonist, these mechanisms require further experimental confirmation.

In human hepatic stellate cells, thrombin and PDGF stimulate the release of PGE_2_, which exerts an inhibitory effect on DNA synthesis induced by PDGF and thrombin [31]. However, PGE_2_ appeared to mediate the mitogenic effect of EGF in BALB/c 3 T3 cells, and of PDGF in Swiss 3 T3 cells [49,50]. In our study, EGF, PGE_2_ and thrombin, but not PDGF, consistently induced COX-2 protein expression in the stellate cells.

Pretreatment of the cells with indomethacin did not affect PDGF-stimulated DNA synthesis, suggesting that COX-2 induction and PGE_2_ production neither mediated nor modulated PDGF-stimulated DNA synthesis. While we did not measure production of PGE_2_, studies in various cells, including pancreatic stellate cells [40], indicate that levels are in the nanomolar range. We did not detect an effect of PGE_2_ on DNA synthesis in the stellate cells when it was added alone, however, PGE_2_, as well as forskolin, inhibited PDGF-stimulated DNA synthesis, suggesting that this effect was mediated by cAMP. This is in contrast to findings in rat pancreatic stellate cells, where treatment of the cells with conditioned medium from the Panc-1 adenocarcinoma cell line induced COX-2 expression and stimulated DNA synthesis [51]. Furthermore, inhibition of COX-2 activity with the COX-2 specific inhibitor NS-398 attenuated DNA synthesis in the rat stellate cells, albeit at high concentrations of the inhibitor, which may lead to nonspecific effects. Thus, at high concentrations of NS-398, inhibition of DNA synthesis has been reported in COX-2 expressing cell lines as well as in cell lines without COX-2 expression [52-54].

Pancreatic stellate cells are believed to be essential in the development of fibrosis associated with chronic pancreatitis and pancreatic cancer [4-6,10]. However, the role of PGE_2_ in pancreatic fibrosis is unknown. TGFβ has been found to induce COX-2, which attenuates the profibrotic effect of TGFβ, in lung fibroblasts and hepatic stellate cells [30,48], and exogenous addition of PGE_2_ inhibited TGFβ-induced collagen expression in hepatic stellate cells [30]. However, we found no induction of COX-2 by TGFβ in the pancreatic stellate cells, and preincubation of the cells with indomethacin did not affect TGFβ-stimulated collagen synthesis. In the lung, PGE_2_ has been found to inhibit collagen synthesis by activating EP2 receptors and stimulating cAMP accumulation. In patients with idiopathic pulmonary fibrosis, lung fibroblasts display a diminished capacity to express COX-2 and to synthesize PGE_2_. This results in decreased levels of PGE_2_ and excessive fibroblast activation with massive fibrosis [41,47,48]. Our findings in the pancreatic stellate cells are consistent with these studies. Treatment with PGE_2_, as well as forskolin, suppressed the increase in collagen synthesis stimulated by TGFβ, suggesting that this effect was mediated by cAMP. Our observations are thus in disagreement with findings in an immortalized human pancreatic stellate cell line, where 100 nM PGE_2_ was found to induce mRNA of collagen 1A1 as well as other structural genes involved in extracellular matrix formation [40]. We therefore examined the effect of PGE_2_ in our stellate cells, and found no evidence of collagen 1A1 mRNA induction. Rather, PGE_2_ (1 μM) attenuated the TGFβ-induced expression of collagen 1A1, which is in agreement with our findings of an inhibitory effect of PGE_2_ on collagen synthesis. The possibility that immortalized pancreatic stellate cells behave differently from primary cell lines needs consideration. Interestingly, the effects of PGE_2_ on immortalized stellate cells were mediated by activation of EP4 receptors [40]. We have found no evidence of EP4 receptor involvement in the cAMP response in our primary stellate cells, however, we can presently not exclude the possibility that EP4 receptors signal via G protein-independent pathways [22].

We observed that PGE_2_ stimulated ERK phosphorylation in the stellate cells. This effect was mimicked by thrombin and the FP selective agonist fluprostenol, but not by forskolin, suggesting that it was a cAMP-independent effect. Thus, the stellate cells may express other EP receptors or FP receptors that mediate this effect. PGE_2_ has been reported to stimulate fibroblast proliferation through activation of EP1, EP3, or FP signalling in lung and cardiac fibroblasts, as well as in NIH 3 T3 cells [26-29]. If other prostaglandin receptors could stimulate proliferation of pancreatic stellate cells, the inhibitory effect of cAMP induced by EP2 receptors, appear to suppress these effects. It is notable that the inhibitory effect of PGE_2_ on collagen and DNA synthesis was only significant at a concentration of 1 μM, whereas in lung fibroblasts effects have been observed at concentrations as low as 10 nM [41]. In a comparative study of fibroblasts from lung and gingiva, it was observed that stimulation with PGE_2_ resulted in less cAMP accumulation in gingival fibroblasts than in lung fibroblasts [55]. Furthermore, EP3 receptor activation induced phosphorylation of c-Jun NH_2_-terminal kinase (JNK), which also mediated TGFβ-stimulated fibrosis. Thus, simultaneous EP3 receptor activation might reduce EP2-stimulated cAMP accumulation and blunt the inhibitory effect on DNA and collagen synthesis. Further studies, using subtype-specific agonists, or knockdown of prostaglandin receptors, are required to explore the role of other prostaglandin receptors on proliferation and fibrosis in the stellate cells.

Several previous studies have demonstrated that COX-2 is overexpressed in most human pancreatic cancers [12-16,56-60]. However, only a few publications have addressed COX-2 expression in pancreatic stellate cells and they reported no detectable COX-2 expression in the stroma [16,60]. In our study, immunohistochemical analysis carried out with a specific monoclonal antibody revealed no detectable COX-2 expression in the stroma – neither in the normal pancreas nor in the pancreatic cancer. In contrast Charo et al. [40] reported COX-2 expression in the stroma. One reason for the discrepancy in the results could be the use of different antibodies. For immunohistochemical staining in the study presented by Charo [40] the polyclonal rabbit antihuman COX-2 antibody was used. It is known that polyclonal antibodies are more sensitive, but do not show as high specificity, as monoclonal antibodies [61]. To confirm the expression of COX-2 in pancreatic stroma, Charo at al [40] performed RT-PCR on isolated stellate cells. However, it is likely that the isolation process itself could cause activation of the stellate cells and increase the COX-2 expression [62].

Expression of COX-2 in cultured pancreatic stellate cells is well documented [40,51,63] and our results support these findings. In the immunofluorescence double staining of the cultured pancreatic stellate cells, only cells with positive expression for αSMA were additionally positive for COX-2. The COX-2 staining was perinuclear and was constant in different passages (data not shown). COX-2 expression could be further induced by stimulating the stellate cells with IL-1β, EGF, thrombin, and PGE_2_. Also, indirect coculture with the BxPC-3 cell line, but not HPAFII or Panc-1 cells, induced COX-2 expression. Pretreatment of the stellate cells with IL-1 receptor antagonist blocked the induction of COX-2 induced by BxPC-3 cells, which is consistent with the fact that the BxPC-3 cell line is known to produce IL-1α [64]. Interestingly, conditioned medium from Panc-1 cells induced COX-2 in rat pancreatic stellate cells, however, how this was mediated was not examined [51].

## Conclusions

The present results show that COX-2 is mainly expressed in carcinoma cells, and suggest that the cancer cells are the main source of PGE_2_ in pancreatic tumours. In the pancreatic stellate cells, PGE_2_ exerts both antiproliferative and antifibrotic effects. These effects of PGE_2_ are mediated by the cAMP pathway and suggests a role of EP2 receptors. Inhibition of COX-2 may inadvertently accelerate fibrosis progression in pancreatic cancer.

## Competing interests

The authors declare that they have no competing interests.

## Authors’ contributions

EP, DS, TC, IPG conceived and planned the study. EP and KG isolated the pancreatic stellate cells. EP and DS did the cell culturing work. EP and ARS did the immunohistochemistry and immunofluorescence work. DS, EP, IHT, MA and VT conducted the experimental work. DS, EP and IPG analysed and discussed the results. DS, EP and IPG drafted the manuscript. All authors read and approved the final manuscript.

## Pre-publication history

The pre-publication history for this paper can be accessed here:

http://www.biomedcentral.com/1471-2407/14/413/prepub
